# The Pharmacokinetics and Pharmacodynamics of Iron Preparations

**DOI:** 10.3390/pharmaceutics3010012

**Published:** 2011-01-04

**Authors:** Peter Geisser, Susanna Burckhardt

**Affiliations:** Research & Development Department, Vifor Pharma – Vifor International Inc, Rechenstrasse 37, St Gallen, CH-9001, Switzerland

**Keywords:** iron, pharmacokinetics, pharmacodynamics, iron complex, metabolism, elimination, kinetics, efficacy, safety

## Abstract

Standard approaches are not appropriate when assessing pharmacokinetics of iron supplements due to the ubiquity of endogenous iron, its compartmentalized sites of action, and the complexity of the iron metabolism. The primary site of action of iron is the erythrocyte, and, in contrast to conventional drugs, no drug-receptor interaction takes place. Notably, the process of erythropoiesis, *i.e.*, formation of new erythrocytes, takes 3–4 weeks. Accordingly, serum iron concentration and area under the curve (AUC) are clinically irrelevant for assessing iron utilization. Iron can be administered intravenously in the form of polynuclear iron(III)-hydroxide complexes with carbohydrate ligands or orally as iron(II) (ferrous) salts or iron(III) (ferric) complexes. Several approaches have been employed to study the pharmacodynamics of iron after oral administration. Quantification of iron uptake from radiolabeled preparations by the whole body or the erythrocytes is optimal, but alternatively total iron transfer can be calculated based on known elimination rates and the intrinsic reactivity of individual preparations. Degradation kinetics, and thus the safety, of parenteral iron preparations are directly related to the molecular weight and the stability of the complex. High oral iron doses or rapid release of iron from intravenous iron preparations can saturate the iron transport system, resulting in oxidative stress with adverse clinical and subclinical consequences. Appropriate pharmacokinetics and pharmacodynamics analyses will greatly assist our understanding of the likely contribution of novel preparations to the management of anemia.

## 1. Introduction

Iron is an essential component of every cell in the body. Although best known for its critical role in the transport and storage of oxygen (in hemoglobin and myoglobin, respectively), within a large variety of enzymes iron also acts as a carrier for electrons, a catalyst for oxygenation, hydroxylation, and is necessary for cellular growth and proliferation. Iron supplements are widely administered to treat iron deficiency anemia, particularly in chronic diseases such as kidney disease [1], heart failure [2] or inflammatory bowel disease [3]. Without a sufficient supply of iron, hemoglobin cannot be synthesized and the number of erythrocytes in the blood cannot be maintained at an adequate level [4]. However, because of the ubiquity of iron, its compartmentalized sites of action, and its complex metabolism, usual pharmacokinetics measurements such as serum concentration are largely irrelevant when evaluating the bioavailability and efficacy of iron preparations [5]. As such, pharmacokinetics and pharmacodynamics assessments of iron preparations cannot be based on the standard principles that apply to non-endogenous drugs.

**Figure 1.**
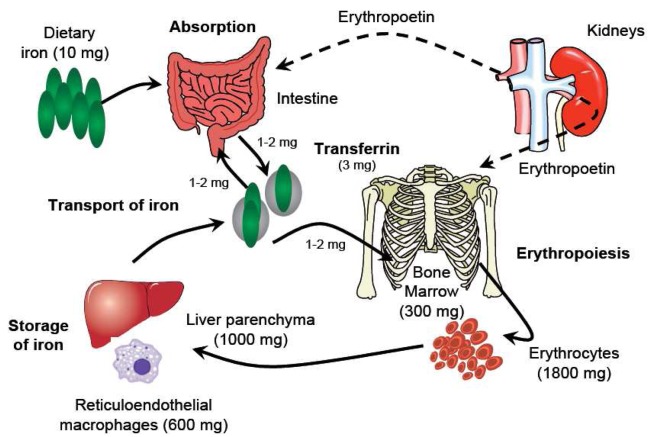
Schematic representation of iron metabolism. Under normal conditions, the iron in the body is in a dynamic equilibrium between different compartments (solid arrows). From approximately 10 mg of iron ingested with food, 1–2 mg are absorbed by duodenal enterocytes and the same amount is lost, e.g., via skin exfoliation. In the circulation, iron is bound to transferrin (*ca.* 3 mg), which safely transports it e.g., to the bone marrow for hemoglobin synthesis. Approximately two-thirds of the iron in the body is found in the form of hemoglobin, in red blood cells (1800 mg) and in erythroid precursors in the bone marrow (300 mg), whereas 10–15% is present in myoglobin and in a variety of different essential enzymes. Iron is stored in parenchymal cells of the liver (*ca*. 1000 mg). Reticuloendothelial macrophages temporarily store the iron recycled from senescent red blood cells (600 mg) in a readily available form. Erythropoetin, produced in the kidneys, regulates duodenal iron absorption and erythropoiesis (dashed lines). Adapted from Crichton, 2008 [7].

Understanding the metabolism of iron underpins any consideration of its pharmacology (Figure 1). Iron usually exists in the ferrous (Fe^2+^) or ferric (Fe^3+^) state, but since Fe^2+^ is readily oxidized to Fe^3+^, which in neutral aqueous solutions rapidly hydrolyzes to insoluble iron(III)-hydroxides, iron is transported and stored bound to proteins. Effective binding of iron is essential not only to ensure that it is available where and when required, but also because Fe^2+^ can catalyze the formation of reactive oxygen species, which cause oxidative stress, damaging cellular constituents. Three key proteins regulate the transport and storage of iron. *Transferrin* transports iron in the plasma and the extracellular fluid. The *transferrin receptor*, expressed by cells that require iron and present in their membranes, binds the transferrin di-iron complex and internalizes it into the cell. *Ferritin* is an iron-storage protein that sequesters iron keeping it in a readily available form. About 60% of iron is found in the erythrocytes within hemoglobin [6], the oxygen transport protein. The remainder is found in myoglobin in the muscles, in a variety of different enzymes (‘heme’ and ‘non-heme’), and in storage form. Most stored iron is in the form of ferritin, found in the liver, bone marrow, spleen and muscles. Serum iron (*i.e.*, iron bound to transferrin) represents only a very small proportion of total body iron (<0.2%) [7]. Moreover, the relationship between physiological iron compartments is highly dynamic: Erythrocytes are broken down in the liver and in the spleen, and new red blood cells are produced in the bone marrow. The total serum iron pool is approximately 4 mg, but the normal daily turnover is not greater than 30 mg [7], such that minor changes in serum level due to exogenous iron administration are clinically meaningless. In this setting, conventional measurements of serum iron concentration provide no relevant information about the availability of functional iron for physiological processes, and other evaluation strategies must be pursued.

## 2. The Pharmacokinetics of Iron

A primary aim of pharmacokinetics analyses is to determine bioavailability, defined by the European Medicines Agency as ‘the rate and extent to which the active substance or active moiety is absorbed from a pharmaceutical form and becomes available at the site of action’ [8]. Typically, bioavailability is assessed based on the serum concentration of the administered product. This model only applies, however, if there is a classical drug-receptor interaction on cell membranes such that efficacy correlates well with the serum concentration of the drug. In the case of iron, the primary site of action is the erythrocyte, with iron storage sites of secondary relevance.

Several definitions have been proposed for iron bioavailability (reviewed in Wienk *et al.* [9]), but the consensus is that it should be a quantifiable measure of the proportion of total iron that is absorbed and metabolized, *i.e.* that is incorporated into hemoglobin [9]. As a consequence, serum concentration is not relevant. Notably, the process of erythropoiesis takes 3–4 weeks [4], such that iron utilization from the time of administration only peaks after approximately 2–3 weeks [10] and short-term area under the curve (AUC) values of serum iron (e.g., over 8 hours) are of much less relevance than long-term (e.g., 3-month) values for iron uptake by erythrocytes. The amount of iron in the serum represents only a small part of the iron that is transferred to the site of action, which is not proportional to the peak serum concentration (C_max_) or to the AUC value but to the rates of transfer and elimination to and from the serum. Thus, other approaches to pharmacokinetics assessment of iron are clearly required [11-13].

### 2.1. Pharmacokinetics of iron after intravenous application

Iron is administered intravenously in the form of iron carbohydrate complexes consisting of a mineral core, composed of polynuclear iron(III)-hydroxide surrounded by the carbohydrate ligand [14]. The main function of the ligand is to stabilize the complex and to protect it against further polynuclearization. Examples include Venofer^®^ (iron sucrose), Ferinject^®^ (ferric carboxymaltose), Ferrlecit^®^ (sodium ferric gluconate in sucrose, for injection) and various iron dextran formulations. Iron carbohydrate complexes of this type behave as prodrugs, since the iron has to be released from the iron(III)-hydroxide core. According to the proposed mechanism, after administration, the stable complexes such as ferric carboxymaltose and iron dextran are taken up by endocytosis by macrophages of the reticuloendothelial system (RES) [14]. In a further step, the endosome fuses with a lysosome and the acidic and reducing environment in the endolysosome leads to cleavage of iron from the complex. The Fe^2+^ generated is transported by the divalent metal transporter 1 (DMT1) across the endolysosomal membrane to enter the labile iron pool within the macrophage cytoplasm. From there, it can be incorporated into ferritin and remain transiently stored within the macrophage or can be transported out of the macrophage by the transmembrane protein ferroportin (as Fe^2+^). The exported Fe^2+^ is immediately oxidized by ceruloplasmin to Fe^3+^ which is sequestered by transferrin for transport in the serum to the sites of utilization, e.g., in the bone marrow for hemoglobin synthesis or in the liver for storage in ferritin.

In the case of less stable preparations, however, this highly regulated process of iron release from carbohydrate complexes can be disrupted. Here, release of significant amounts of labile iron from the complex can lead to saturation of transferrin and, thus, to significant amounts of non-transferrin bound iron (NTBI), particularly if high doses are administered. This weakly bound Fe^3+^ is readily taken up in an unregulated way by cells of the endocrine system, the heart, and the liver, where it can induce oxidative stress by catalyzing lipid peroxidation and reactive oxygen species formation [15].

In general, complexes can be classified as labile or robust (kinetic variability, *i.e.* how fast can ligands coordinated to the iron be exchanged) and weak or strong (thermodynamic variability, *i.e.* how strongly are the ligands bound to the iron, and thus, how much energy is required to dissociate a ligand from the iron), or any intermediate state (Table 1) [7]. The reactivity of each complex correlates inversely with its molecular weight, *i.e.* larger complexes are less prone to release significant amounts of labile iron or react directly with transferrin [14,17]. Type I complexes such as iron dextran preparations (Imferon^®^, Cosmofer^®^, InFeD^®^, Dexferrum^®^) or ferric carboxymaltose (Ferinject^®^) have a high molecular weight and a high structural homogeneity, and, thus, deliver iron from the complex to transferrin in a regulated way via macrophages endocytosis and subsequent controlled export [7,10]. Such complexes can be administered intravenously and are clinically well-tolerated even at high doses [17]. Type II complexes (iron sucrose complexes such as Venofer^®^) are semi-robust and moderately strong, and release larger amounts of weakly bound iron in the blood. Thus, larger amounts of iron are taken up directly by transferrin and other proteins, and only the iron core is taken up via endocytosis by the macrophages of the reticuloendothelial system. Despite the lower molecular weight and complex stability compared to Type I complexes, Type II complexes are still suited for intravenous application. Nevertheless, the maximal single doses are significantly lower and the administration times drastically longer. Type III and IV complexes, including sodium ferric gluconate (Ferrlecit^®^) and iron(III)-citrate + iron(III)-sorbitol + iron dextrin (Jectofer^®^), have variable amounts of low molecular weight components (<18,000 Daltons) and are characteristically labile and weak [17]. In general, intravenous use of preparations containing large amounts of complexes with a molecular weight below 18,000 Daltons should only be undertaken with care [17]. These types of iron complexes are likely to generate large amounts of NTBI, which may then bind to various types of proteins – only if they are administered in small doses is the iron taken up primarily by macrophages (endocytosis). Moreover, all iron complexes with molecular weight below 18,000 Daltons are subject to undesirable renal elimination [17].

**Figure 2.**
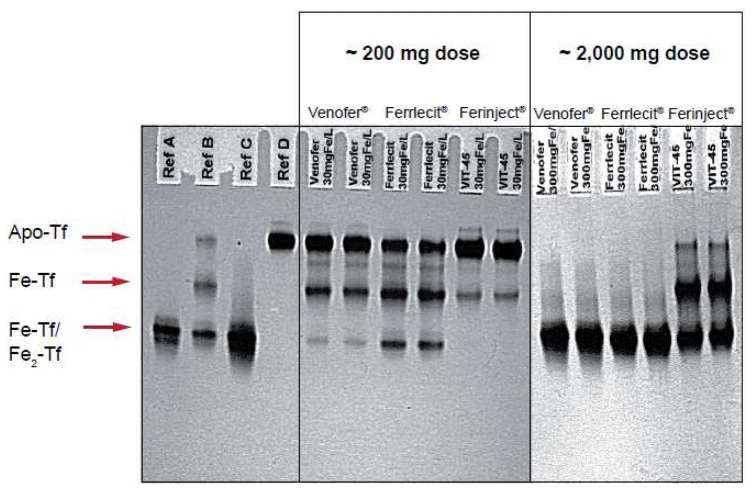
*In vitro* reactivity of Ferinject^®^, Venofer^®^ and Ferrlecit^®^ with apotransferrin. Urea polyacrylamide gel electrophoresis (PAGE) of transferrin incubated with different amounts of various intravenous iron preparations. Apo-Tf, transferrin with no iron; Fe-Tf, transferrin with one iron-binding site occupied; Fe_2_-Tf, transferrin with both iron-binding sites occupied [holotransferrin]. The reactivity towards apotransferrin was the lowest with the most stable complex, *i.e.* Ferinject^®^. At concentrations equivalent to those expected in the serum of an adult after a therapeutic dose of ∼200 or ∼2,000 mg of iron, transferrin saturation was observed with Ferrlecit^®^ and Venofer^®^ but not with Ferinject^®^ (Technical communication, Vifor Pharma – Vifor International Inc).

Figure 2 illustrates the results of an *in vitro* study that compares the relative reactivity of Ferinject^®^, Venofer^®^ and Ferrlecit^®^ towards apotransferrin. In this experiment, apotransferrin was incubated with different amounts of the three intravenous iron preparations at a final concentration equivalent to that expected in the serum of an adult patient after injection of ∼200 or ∼2,000 mg of iron. It is noteworthy that Ferinject^®^ has a significantly lower reactivity than the two other complexes. Even at a dose equivalent to ∼2,000 mg iron, Ferinject^®^ does not induce full saturation of transferrin. Weakly bound low molecular weight components result in transferrin saturation and the consequent oxidative stress induced by NTBI leads to adverse events such as hypotension, nausea, vomiting, abdominal and lower back pain, peripheral edema and a metallic taste [19].

**Figure 3.**
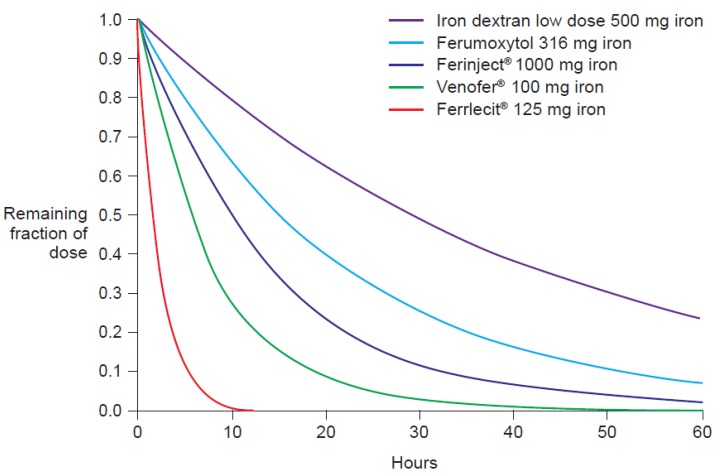
Normalized simulated single first-order elimination kinetics for different intravenous iron preparations, depicted as fraction of total serum iron over time. Values of the terminal elimination rates given in Table 2 were used to calculate an overall first-order kinetics and t_1/2_ values. The figure clearly shows that the AUC is negatively correlated to the elimination rate constants.

The molecular weight of the intravenous iron carbohydrate complexes strongly influences not only the rate of release of iron from the core but also the rate of clearance from the plasma [14]. In fact, Type I complexes have a long half-life of elimination, e.g., Ferinject^®^ 7–12 hours and iron dextran 1–3.5 days (dose-dependent), compared to an elimination half-life of 5–6 hours for iron sucrose (Venofer^®^) [20] and <4 hours for Types III and IV [7] (e.g. Ferrlecit^®^ 1–1.5 hours [21]). The pharmacokinetics parameters of different intravenous iron preparations have been measured in separate Phase I studies under similar conditions (Table 2) [20–22,24,25]. Based on these parameters, we calculated the normalized AUC after intravenous application of a dose of 100 mg iron for the various iron carbohydrate complexes (Table 2). The results clearly show that AUC is strongly influenced by the terminal elimination rate, which is dependent on the molecular weight of the complex, and not by the dose (Table 2). Moreover, the standardized elimination curves depicted in Figure 3, calculated based on the values of the terminal elimination rates given in Table 2, clearly show the negative correlation between AUC and the elimination rate constants.

Thus, mean serum concentration and AUC do not increase linearly with the dose of injected iron but are inversely correlated with the elimination rates [22,26]. Examination of the total serum iron concentration curves after intravenous application revealed that the elimination of iron from the serum can be explained with an overlap (superimposition) of a zero-order (constant rate) and a first-order elimination function [14,20,24]. This model explains the non-linear relation between the administered dose and the AUC value [12]. By using an open two-compartment model system with an underlying baseline level as well as an underlying Michaelis-Menten term, the serum iron level can be calculated according to the following formula [20]:

$$\text{C}(\text{t})={\text{ae}}^{-\alpha \text{t}}+{\text{be}}^{-\beta \text{t}}+{\text{C}}_{\text{B}}-{\text{k}}_{0}\text{t}$$

where C(t) is the time-dependent serum iron concentration, a, b, α and β are hybrid constants, C_B_ is the iron pre-dose level and k_0_t is the Michaelis-Menten term. The final distribution volume is normally about 3 liters for a 70 kg person. With the help of k_0_, the amount of iron taken up by macrophages and/or the iron transferred by transferrin to other compartments can be calculated. From the dose (D) and the difference between the first post-dose C_0_ and pre-dose level C_B_, the volume of distribution of the central compartment V_c_ can be determined.

### 2.2. Pharmacokinetics of iron after oral application

Absorption of iron from the gut is carefully regulated. Because there is no active excretory process for iron once it has entered the bloodstream, the body's control of iron levels is undertaken at the level of the enterocyte. Iron in food, in the form of Fe^3+^, is reduced to Fe^2+^ by duodenal cytochrome *b* (Dcyt *b*) in the enterocyte membrane then imported by DMT1 into the enterocyte cytoplasm, where it can either be stored as ferritin or be exported to the serum via the basolateral transport protein ferroportin [27]. This export protein is coupled to multicopper oxidases (hephaestin in the membrane or ceruloplasmin in the serum), which oxidize Fe^2+^ to Fe^3+^, which finally is tightly bound to transferrin [27]. The mechanism of uptake of heme iron, derived from meat, is not well understood. It has been proposed that the enterocyte membrane also contains a protein that can transport heme iron from the gut lumen into the cytosol (HCP1) [28]. However, the same protein has later been shown to be responsible for folate transport in the intestine, with a significantly higher affinity [29–31]. In the enterocyte, Fe^2+^ is released from the heme in a process catalyzed by heme oxygenase [32] and enters the same cytosolic pool as non-heme iron.

A typical diet contains approximately 10–20 mg iron/day, but the fixed-rate physiological uptake route allows for absorption of only up to 5 mg at a time [13,33]. A therapeutic oral iron dose of, for example, 100 mg, thus largely exceeds the amount that can be taken up via the active absorption pathway. Due to the physico-chemical properties of ferrous salts, passive uptake occurs through the paracellular route [33] such that a portion of the Fe^2+^ in the gut is absorbed directly by the blood. Under normal circumstances, transferrin in the blood is approximately one-third saturated [7]. However, under the pressure of passive diffusion, transferrin becomes saturated and NTBI circulates in the plasma, is taken up via an unregulated mechanism by endocrine and heart cells, resulting in oxidative stress reactions within these tissues. With rapidly absorbed preparations, NTBI can be observed even before transferrin is fully saturated.

**Figure 4.**
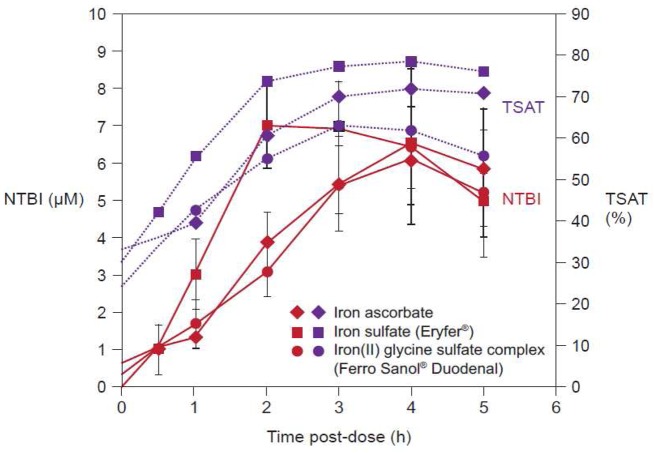
Serum concentration of non-transferrin bound iron (NTBI) and percentage transferrin saturation (TSAT) following administration of a single oral dose of 100 mg iron in the form of three different ferrous salts to healthy adult volunteers. Broken blue lines indicate the percentage transferrin saturation (right-hand axis). Solid red lines indicate NTBI concentration (left-hand axis). Values shown are mean ± SD. Modified from Dresow *et al.* 2008 [34].

**Figure 5.**
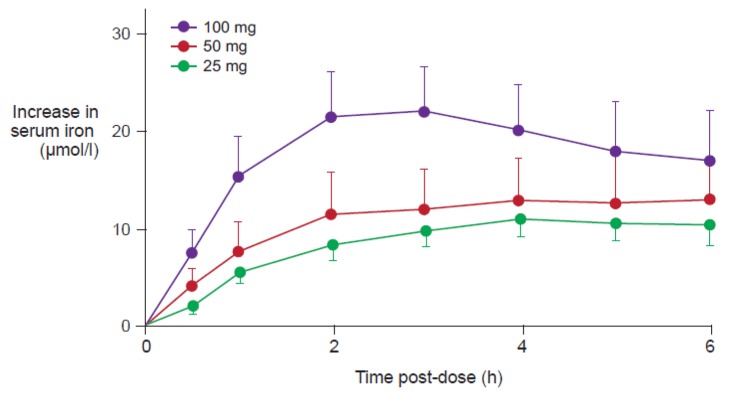
Increase in serum iron concentration after administration of 25, 50 and 100 mg ferrous iron in 6 healthy subjects [37]. Data are shown as mean ± SEM. The data clearly show that there is no linear relationship between serum iron increase (C_max_ and AUC) and dose.

Figure 4 illustrates the quantification of NTBI in serum samples from adult volunteers with normal iron stores after oral administration of 100 mg iron in the form of ferrous salts [34]. NTBI concentrations of up to 9 μM were observed within the first four hours post-dose even though transferrin saturation (TSAT) was below 100%. Significant levels of NTBI were detected even at lower doses, e.g., 10 mg iron as ferrous ascorbate or ferrous glycine sulfate [34]. In the same study, it was reported that iron(III)-polymaltose at a dose of 150 mg iron resulted in a maximal NTBI concentration of only 0.7 μM, close to the detection limit of the assay that was used [34]. Interestingly, a similar study showed that significant levels of NTBI are also produced when oral iron preparations based on ferrous salts are taken with food [35]. As the iron dose given in the form of ferrous salts increases, the proportion of iron absorbed through the passive paracellular route increases, such that NTBI rises [34], consistent with the dose-related nature of side effects associated with oral iron therapy [36]. Even passive absorption, however, can become saturated such that ever-increasing doses of oral iron do not result in proportionately higher AUC, a finding demonstrated by Ekenved and coworkers following administration of 25, 50, and 100 mg iron as ferrous sulfate solution (Figure 5) [37]. A linear pharmacokinetics model can therefore be excluded [13]. Thus, a maximum serum iron increase of, for instance, 20 μmol/L can correspond to intestinal iron absorption of between 3.5 and 17 mg [37,38].

If results from other studies are used, this variance will increase even more [39]. In contrast, Heinrich *et al.* [40] reported a somehow better correlation between iron absorption and the serum iron concentration measured 3 h after a dose of 100 mg iron on an empty stomach. However, the conclusion of the authors is that the serum iron measurement gives only semi-quantitative information on the bioavailability of therapeutic iron preparations [40]. Notably, Heinrich and coworkers included iron(III)-preparations (ferric citrate and iron(III)-polymaltose) in their study, despite the fact that it is known that the absorption of these preparations is up to seven-times better when taken with food [41] and thus cannot be compared under the same conditions (e.g., empty stomach).

The serum concentration of iron following oral administration is strongly dependent on both invasion and elimination kinetics. As with intravenous administration, iron elimination after oral iron application can be fitted with a zero-order function [13]. More rapid absorption from a given preparation results in larger serum AUC and higher maximal serum iron concentration, since the AUC strongly depends on the invasion kinetics because the zero-order elimination rate is the rate-limiting step [13]. Since the rate of transfer and the time for serum iron to return to baseline are both constant, AUC values do not reflect the true extent of iron absorption and AUC shows no correlation with erythrocyte uptake following oral iron administration [42]. Since high serum concentration of iron can result in NTBI, with the associated risk of oxidative stress and related adverse effects, a more rapid absorption rate is in fact disadvantageous.

In an attempt to reduce the adverse events of ferrous salts, more slowly absorbed preparations have been developed. Ferrous fumarate, the least toxic iron(II) compound, causes fewer adverse events because of its low solubility and slow dissolution rate after oral administration [7]. In effect, the rate of release of ferrous ions from ferrous fumarate is slower than that from the highly soluble ferrous sulfate.

One of the available ferrous fumarate formulations on the market is Ferrum Hausmann^®^ capsules. Geisser *et al.* examined the pharmacokinetics and bioavailability of standard ferrous fumarate and this slow-release formulation in a randomized study of 20 healthy volunteers with depleted iron stores. Results demonstrated that the two preparations were bioequivalent despite slower absorption of iron and lower AUC values with the slow-release formulation [13]. Kaltwasser *et al.* have confirmed that standard or slow-release preparations (in this case, ferrous sulfate) exhibit similar iron bioavailability [43].

The pharmacokinetics profile of iron following oral administration of iron(III)-polymaltose complex is quite different from that of ferrous salts. The iron(III)-polymaltose complex is made of non-ionic iron(III), in a form of polynuclear iron(III)-hydroxide, and polymaltose ligands. The resulting complex is stable. Being in a non-ionic form, iron does not interact with food components and does not induce the generation of reactive oxygen species.

Pharmacokinetics of the iron(III)-polymaltose preparation Maltofer^®^ have been extensively studied. During the first six hours after administration of Maltofer^®^, only a negligible increase in serum iron concentration is observed, *i.e.*, as expected from the size of the complex, there is virtually no passive diffusion through intercellular spaces [44]. Nevertheless, 2–3 weeks after application of Maltofer^®^ the incorporation of iron into erythrocytes is not significantly different to that seen with ferrous salts [44]. Similar bioavailability of iron following administration of Maltofer^®^, ferrous sulfate or ferrous fumarate has been confirmed by other authors [45], as well as comparable hemoglobin increase by using Maltofer^®^ or ferrous sulfate at the same dose (100 mg iron twice a day) [46]. Interestingly, iron absorption from Maltofer^®^ appears to be enhanced in the presence of food, in contrast to the situation with oral ferrous salts where absorption is diminished. As with simpler preparations, there is no correlation between AUC and bioavailability measured by erythrocyte uptake of iron [42] and thus, measurements of serum iron AUC are of no relevance for estimates of efficacy of oral iron(III)-polymaltose complex.

### 2.3. Pharmacokinetics of iron: conclusions

Extensive pharmacokinetics analyses and the understanding of the delivery pathways of iron to relevant physiological compartments demonstrate that the serum iron concentration or the AUC measured following iron supplementation cannot be used to assess efficacy of iron preparations. In particular, the kinetics of iron absorption depend on the type of oral iron preparation: compounds that are absorbed slowly inevitably lead to lower maximal plasma iron increases, smaller AUC, and consequently to misinterpretation of the results [9]. Thus, in the case of iron therapy, these conventional pharmacokinetics markers do not offer a meaningful estimate of bioavailability in terms of iron utilized within the erythrocyte for hemoglobin synthesis or the amount of iron incorporated in the storage protein ferritin.

Rapid iron absorption and/or high doses of oral preparations can saturate the regulated active absorption mechanisms in the intestine, leading to passive absorption, saturation of the transport protein (transferrin) and generation of weakly bound Fe^3+^ (NTBI), which can induce oxidative stress. This is highly relevant as during oral iron therapy patients usually take 2–3 tablets a day for several months and are thus exposed to oxidative stress on a daily basis for a prolonged time.

## 3. The Pharmacodynamics of Iron

The pharmacokinetics profiles of iron preparations can provide useful information regarding reactivity with transferrin, the risk of adverse events, and offer guidance on possible dosing regimen. To understand and predict the bioavailability of such preparations, however, a more detailed investigation is required. Several experimental approaches to pharmacodynamics analyses allow assessment and comparison of iron absorption and, thus, the efficacy of different preparations [9].

### Radiolabeling techniques

**Figure 6.**
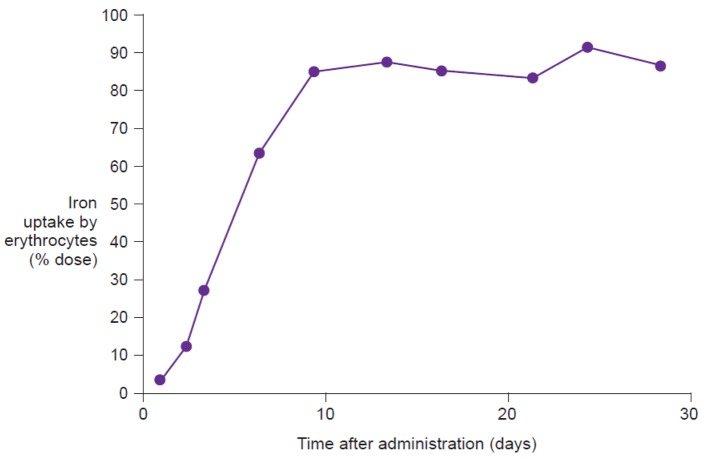
Utilization of iron following a single intravenous administration of radiolabeled iron sucrose (Venofer^®^) in a patient with iron deficiency anemia (modified from Beshara *et al.* 1999 [10]).

Measurement of the uptake of radioactive isotopes (e.g., ^59^Fe), either in the whole body or only in the erythrocytes, represents the reference method for assessing iron bioavailability [9]. Whole body counting determines the total amount of labeled iron retained in the body, including iron temporarily stored in the reticuloendothelial system or deposited in liver ferritin, and as such is the most comprehensive measurement of iron utilization. Erythrocyte counting represents a good measure of how much administered iron is utilized for erythropoiesis. In highly iron-deficient anemic individuals, virtually all absorbed iron will be delivered to the erythrocytes, and thus, for this population, erythrocyte counting offers a good estimation of iron utilization (Figure 6). Evaluation on day 14 after administration of the labeled compound, adjusted for radioactive decay, allows time for incorporation of the isotope into erythrocytes [47]. Thus, erythrocyte iron utilization is usually expressed as a percentage fraction of iron recovered in the cell mass on day 14 after intake. Indeed, Potgieter *et al.* have confirmed that there is a close correlation (r^2^ = 0.91) between ^59^Fe uptake by the erythrocytes and by the whole body following administration of oral iron(III)-polymaltose (Maltofer^®^) [42].

Jacobs *et al.* developed a twin-isotope technique to compare the bioavailability of two different iron preparations, whereby each individual receives two preparations labeled with different iron isotopes (^55^Fe or ^59^Fe) and acts as a self-control [45,48]. Using this technique, the group has shown that iron availability is equivalent following oral administration of either ferrous sulfate or iron(III)-polymaltose (Maltofer^®^) at both physiological and therapeutic doses [45].

### Stable isotope labeling techniques

Because of ethical concerns regarding the use of radiolabeled isotopes, in particular in children, stable iron isotopes (^57^Fe or ^58^Fe) are often used to assess the bioavailability of iron preparations. The amount of labeled iron absorbed can be calculated from the shift in the iron isotopic abundance in the blood after incorporation in red blood cells, approximately 14 days after administration. The different iron isotopes can be measured by inductively coupled plasma mass spectrometry (ICP-MS) [49,50].

### Calculation of total iron transfer

Following oral iron supplementation, the total amount of iron transferred to the iron metabolic pathway (*i.e.*, the true bioavailability) can be calculated from the serum iron concentration because the elimination kinetics for iron primarily follows a zero-order rather than a first-order function due to the fixed-rate reactivity with transferrin. An open two-compartment model system with an underlying baseline level as well as an underlying Michaelis-Menten (MM) term can be applied as follows [7]:

$$\text{C}(\text{t})=\text{a}\;(1-{\text{e}}^{-\text{kin}*\text{t}})-{\text{k}}_{0}\text{t}$$

where C(t) is the serum iron concentration at time t, a is a constant, k_in_ is the rate constant for iron absorption from a particular compound, and k_0_ is the rate constant for elimination (*i.e.* the saturated iron transfer process). Thus, k_0_t is the MM term. Because transferrin is readily saturated with iron (*i.e.* zero-order kinetics) and since the pre-dose serum iron concentration is not statistically significantly different from that measured 24 hours after injection, the MM term v_max_ (the maximum elimination rate) can be regarded as equivalent to k_0_t, where v_max_ reflects the maximal rate of transfer by transferrin. From this term, one can calculate the total amount of iron transported by transferrin during the 24-hour observation period [20].

**Figure 7.**
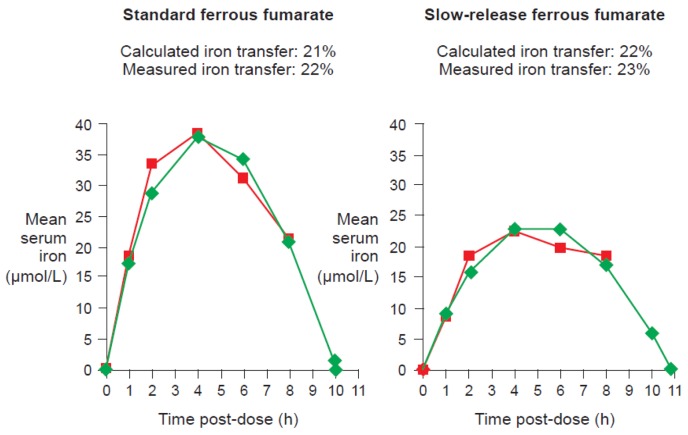
Illustration of the mean measured serum iron concentration (red lines) and the calculated curve (green lines) based on the following equation: C(t) = a (1−e^−kin^*^t^) − k_0_t, where C(t) is the serum iron concentration at time t, a is a constant, k_in_ is the rate constant for iron absorption, and k_0_ is the rate constant for elimination. Data are from an open-label, single-dose, randomized, crossover bioequivalence study in 20 healthy female volunteers given standard oral ferrous fumarate or slow-release ferrous fumarate at a dose equivalent to 100 mg iron per intake (Modified from Geisser *et al.*, 2009 [13]).

Data from studies of ferrous fumarate [13] and ferrous sulfate [43] have confirmed that there is a close correlation between measured iron transfer to erythrocytes and the value calculated from the curve based on this equation for either standard or slow-release formulations (Figure 7).

Because iron shows first-order invasion and zero-order elimination kinetics, the total iron transfer can then be estimated (*i.e.* bioavailability) based on elimination rate constants (Table 2), by using the following equation [13]:

$$\text{Total}\;\text{iron}\;\text{transfer}\;(\text{mmol})={\text{k}}_{0}\;(\text{mmol}/\text{L}/\text{h})\times \text{t}\omega (\text{h})\times {\text{V}}_{\text{d}}\;(\text{L})$$

where k_0_ is the elimination MM constant, tω is the time for total serum iron to reach baseline after administration (which is a finite time in the case of zero-order elimination) and V_d_ is the volume of distribution. This equation is far more informative about the bioavailability of iron than the serum AUC value since it permits calculation of the transfer of iron to compartments (notably erythrocytes) based on serum iron concentration over time, on the assumption that all iron transported by transferrin is delivered to the erythrocytes. Radiolabeled iron experiments have shown that the iron transfer calculated with this equation corresponds closely to the measured concentration of radiolabeled iron taken up by erythrocytes [43,51]. Kaltwasser *et al.* assessed iron pharmacokinetics and iron availability in erythrocytes using stable ^54^Fe in healthy male volunteers given 160 and 150 mg iron daily in the form of a standard-release and a slow-release preparation of oral ferrous fumarate [43]. Based on their data, the total iron transfer calculated with the formula above is 21 and 22% of the administered dose (31 and 34 mg, respectively) – very similar to the 22 and 23% measured by radiolabeled-iron uptake in the erythrocytes [13]. The reliability of the total iron transfer equation has also been shown when applied to iron absorption data obtained by Hallberg *et al.* [51]. Here, the calculated iron transfer was 7.26 mg iron compared to 6.93 mg based on radiolabeled iron measurement in the erythrocytes [13].

The robustness of the transfer calculation means that where expensive isotope technique measurements cannot be undertaken, a close estimate of the amount of iron transferred from an oral preparation to compartments can be made based on total serum iron data over time upon administration of standard-release or slow-release formulations. However, this approach cannot be applied to Maltofer^®^ because serum iron levels are too low to be measured accurately.

### Convolution integral technique

A convolution integral technique has been proposed for calculation of intestinal iron absorption, by which simultaneous administration of differently-labeled oral and intravenous iron doses are used to calculate the iron influx rate into the plasma, and the efflux rate out of the plasma, from which the cumulated intestinal absorption can be summed up [52]. This strategy can only estimate iron bioavailability from single doses, and is less accurate than transfer calculations.

### Fecal monitoring

This method is based on a comparison of all nutritional and medicinal iron intake *versus* the total amount of iron in stools over a fixed time period. This period needs to be at least two weeks due to storage of iron in the gut wall, which can prolong excretion of orally administered iron. In contrast to many drugs, since there is not an active iron excretion pathway, iron loss is restricted to the feces if bleeding from all sources is excluded. Due to the inherent practical difficulties and inaccuracy of this approach, and the very small difference in iron intake *versus* excretion, the results of fecal monitoring are questionable and this approach is rarely used [11].

### Hemoglobin repletion

In the presence of profound iron-deficiency anemia, almost all iron in the serum is transferred to the bone marrow for hemoglobin synthesis and essentially none is stored in the storage protein ferritin. Under these circumstances, the bioavailability of iron can be estimated from the increase in hemoglobin concentration in the blood, using a fixed iron content of 3.47 mg iron/g hemoglobin [11]. This estimate can only be regarded as even approximately reliable, however, if there is no detectable storage iron (serum ferritin concentration <10 ng/mL) and pre-treatment hemoglobin is <10 g/dL with iron supplementation >50 mg iron/day for 2–4 weeks, and if there are no blood or other iron losses – conditions that are only likely to be met infrequently.

## 4. Safety of Iron Preparations

If transferrin is saturated due to rapid release of large amounts of iron from intravenous preparations or high-dose oral iron therapy with ferrous salts, NTBI (weakly bound Fe^3+^) in the serum is rapidly taken up by endocrine and heart cells in an uncontrolled way and, in these tissues, readily participates in reactions that catalyze reactive oxygen species formation and thus promote lipid peroxidation, membrane disruption, enzyme inactivation, sulfhydryl oxidation, and DNA strand breakage and ultimately organ malfunction [7,15,53]. As a consequence, systemic adverse events including hypotension, nausea, abdominal and lower back pain, peripheral edema and a metallic taste can develop [19] and may occur after oral iron supplementation with ferrous salts or intravenous administration of low molecular weight iron complexes. In addition, local reactions in the gut induced by reactive oxygen species produced by reactions initiated and catalyzed by ferrous ions may induce symptoms such as vomiting, dyspepsia, diarrhea and heartburn in ∼20% of patients [36,54]. Finally, it has also been shown that iron (for example in the form of ferric citrate) down regulates expression of CD4 on the surface of T-lymphocytes [55,56], leading to a transient impairment of immunological defenses.

### 4.1. Safety of intravenous iron preparations

For intravenous preparations, the rate and the extent of release of weakly bound iron is inversely related to the size of the molecule, with degradation rates increasing progressively from iron dextran, to ferric carboxymaltose (Ferinject^®^), iron sucrose (Venofer^®^) and sodium ferric gluconate (Ferrlecit^®^) [17]. Type I preparations, such as iron dextran and ferric carboxymaltose, bind iron tightly as non-ionic polynuclear iron(III)-hydroxide and do not release large amounts of iron ions in the blood. Thus, they are clinically well-tolerated even when administered at high doses. In a pooled analysis of 10 randomized trials involving approximately 2,800 patients with iron-deficiency anemia, no treatment-related serious adverse events were observed among Ferinject^®^-treated patients, and there was a markedly lower rate of adverse events than oral iron (primarily ferrous sulfate): 15.3% *versus* 26.1%, respectively [57]. In a recent randomized trial of 459 iron-deficient patients (with and without anemia) with chronic heart failure, there was a similar rate of adverse events as well as serious adverse events in the Ferinject^®^ and the placebo cohorts [58], a result that reflects the stability of the ferric carboxymaltose complex.

Moreover, all iron complexes that contain dextran can lead to dextran-induced, potentially fatal anaphylactic reactions due to specific interaction with dextran antibodies [59]. Anaphylaxis has been reported in 0.60% of patients receiving intravenous iron dextran [60]. Such reactions can occur even with iron preparation with derivatized dextran ligands or low molecular mass dextran ligands (1,000–7,000 Daltons), although less frequently [7,61]. Indeed, rare cases (0.2%) of anaphylaxis or anaphylactoid reactions have been reported with ferumoxytol (Feraheme^®^), a new intravenous iron formulation with carboxymetylated dextran [62]. Notably, a recent case highlighted the risk of anaphylaxis following treatment with ferumoxytol in patients with a history of hypersensitivity to iron dextran [63]. In contrast, anaphylactic reactions are highly unlikely with dextrin- or sucrose-containing complexes such as ferric carboxymaltose and iron sucrose because dextrin and sucrose do not react specifically with dextran antibodies. Indeed, no anaphylaxis has been reported upon administration of ferric carboxymaltose.

The lower molecular weight of Type II-IV preparations can be considered an advantage over the Type I complexes in terms of potential anaphylactic reactions. Since Type II preparations contain no biological polymers, serious adverse reactions would be expected to be less frequent than with iron dextran. Indeed, large-scale post-marketing data on iron sucrose have reported only 4.2 adverse events per million 100 mg iron dose equivalents (compared to 29.2 for iron dextran) [60]. True anaphylactic reactions cannot occur with iron sucrose or sodium ferric gluconate, although very occasionally adverse events triggered by weakly bound iron have been reported [59], in particular when higher than recommended doses are administered. However, smaller and more labile Type III and IV iron complexes, with significant amounts of components with a molecular weight <18,000 Da, cannot be regarded as clinically safe when applied intravenously [7]. Sodium ferric gluconate, even at relatively low doses has been shown in nonclinical studies to result in severe and extended parenchymal liver necrosis secondary to lipid peroxidation induced by the iron [17]. The rate of adverse events reported to the Food and Drug Administration (FDA) in patients receiving sodium ferric gluconate is approximately double that of iron sucrose [60]. Iron(III)-citrate and iron(III)-sorbitol are very rapidly eliminated by the kidneys, such that only small amounts are deposited in the liver. However, nonclinical studies show that iron overload is detectable in the kidney tissue for a limited time after administration [17].

The long-term safety of intravenous iron preparations is a matter of discussion. However, recent comprehensive reviews have come to the conclusion that, in particular with the new intravenous iron preparations, these concerns are unfounded [64]. One potential safety issue is linked to the eventual long-term storage of complexes due to the non-complete utilization of iron from intravenous preparations. This concern is based on the observation that, most likely because of the very high stability of the iron dextran complexes, the utilization of iron from these preparations is not quantitative [65]. In contrast, the comprehensive utilization experiments carried out with radiolabeled iron in the form of iron sucrose [10] and ferric carboxymaltose [47] showed that in patients with iron-deficiency anemia, the utilization of iron from these complexes is essentially quantitative. Detailed studies in this form are not available for sodium ferric gluconate or ferumoxytol, for which the question of quantitative utilization remains unanswered.

### 4.2. Safety of oral iron preparations

Different oral preparations exhibit different safety profiles, with ferrous sulfate—the cheapest and most commonly prescribed oral iron supplement—showing a rapid rise in both serum iron concentration and NTBI [35] and the greatest frequency of adverse events [36,54]. Overall, greater oxidative stress is observed with oral iron(II) salts than with orally administered iron(III) complexes due to more rapid release of iron ions. Toumainen *et al.* carried out a six-month, double-blind study in 45 men with low iron stores, given either ferrous sulfate (180 mg iron a day) or iron(III)-polymaltose (Maltofer^®^, 200 mg iron a day) [66]. Oxidative susceptibility, as measured by low density lipoproteins, was 12.8% higher in the ferrous sulfate group compared to the Maltofer^®^ group; the amount of lipid peroxidation products was 13.8% higher.

These data are consistent with the findings of a single-center, open, randomized, multidose study in which equivalent doses (100 mg iron twice a day for 12 weeks) of iron(III)-polymaltose complex (Maltofer^®^) and ferrous sulfate were administered to anemic volunteer blood donors [46]. At the end of the trial, the improvement in hemoglobin concentration was comparable in the two groups. However, adverse events were markedly less frequent in this group (12.5% of subjects compared to 44.7% in the ferrous sulfate group). A number of studies have observed a lower rate of treatment interruption with iron(III)-polymaltose complex (Maltofer^®^) than with ferrous salts, usually as a result of fewer upper gastrointestinal tract adverse events [67]. Thus, given the similar bioavailability of Maltofer^®^ and ferrous salts, the slower absorption of iron from the non-ionic iron(III)-polymaltose complex is preferable to standard-release oral preparations in terms of the efficacy/toxicity balance.

## 5. Conclusions

Conventional pharmacokinetics analyses are uninformative about iron bioavailability following administration of oral iron preparations. Pharmacokinetics evaluation can elucidate absorption and transport processes, and provide an indication of the relative risk of adverse events, but are irrelevant for efficacy assessment since the biological site of action for iron therapy is the erythrocyte, not the serum. Thus, measurements of serum transferrin concentration or serum iron AUC cannot be applied in the setting of iron therapy and more sophisticated pharmacodynamics analyses must be pursued to obtain meaningful data on the efficacy of a given iron preparation. These strategies are based on calculating the concentration of iron in the key physiological compartment – the erythrocyte. Ideally, pharmacodynamics assessment should be carried out by isotope studies, but if this is impractical or financially prohibitive, iron transfer calculations offer a reliable alternative for assessment of ferrous salts preparations. When the blood transport system, *i.e.* transferrin, becomes saturated, for example with a high intravenous dose of a labile or semi-robust iron complex or with a rapid-release oral ferrous salt preparation, transferrin saturation results and non-transferrin bound iron induces oxidative stress with consequent clinical and subclinical adverse events. Indeed, the frequency and severity of adverse events is highly dependent on the amount of non-transferrin bound iron.

Currently, a number of iron preparations are in development and this expansion is likely to continue [59]. Applying appropriate pharmacokinetics and pharmacodynamics will greatly assist our understanding of the likely contribution of novel preparations to the management of anemia.

## Figures and Tables

**Table 1.** Classification of intravenous iron carbohydrate complex preparations [17].

	**Type I**	**Type II**	**Type III**	**Type IV**
Example	Ferric carboxymaltose Iron dextran Ferumoxytol	Iron sucrose	Sodium ferric gluconate Iron(III)-citrate Iron(III)-sorbitol	Iron(III)-citrate + iron(III)-sorbitol + iron dextrin Sodium ferric gluconate + iron sucrose
Preparations	Ferinject^®^* InFeD^®^ Cosmofer^®^ Imferon^®^ Dexferrum^®^ Feraheme^®^	Venofer^®^ Fesin^®^		Jectofer^®^ Ferrlecit^®^
Characteristics	Robust Strong	Semi-robust Moderately strong	Labile Weak	Mixtures containing at least two different iron complexes
Molecular weight (Daltons)	> 100,000	30,000–100,000	<50,000	<50,000
*In vitro* degradation kinetics (k × 10^3^ /min at θ = 0.5) [16]	15–50	50–100	>100	>100
*In vitro* percentage iron donation to transferrin (%) [18]	2.4 - 3.4 (iron dextran)	4.5 (Venofer^®^)	Not available	5.8 (Ferrlecit^®^)
LD_50_ (mg iron/kg)	1,013 (iron dextran)	359 (Venofer^®^)	Not available	155 (Ferrlecit^®^)

* Injectafer^®^ in some markets; LD_50_ in white mice.

**Table 2.** Pharmacokinetics parameters for intravenous iron preparations.

**Parameter**	**Ferrlecit^®^ Sodium ferric gluconate**	**Venofer^®^ Iron sucrose**	Ferinject^®^* Ferric carboxymaltose	**Imferon^®^ Iron dextran USP/BP**	**Feraheme^®^ Ferumoxytol**
Molecular weight (Dalton)	37,500^1^ 200,000^2^	43,300^1^ 252,000^2^	150,000^1^ not measured	103,000^1^ 410,000^2^	185,000^1^ 731,000^2^
Reactivity with transferrin	High	Medium	Low	Low	Low
Dosage used for the following PK characteristics, mg Fe	125^3^	100^4^	100 / 1,000^5^	500–2,000^6^	316^7^
terminal k_el_, h^−1^	0.488	0.145	0.094 / 0.074	0.024^6^	0.048
k_0_, mg Fe/L*h	-	0.1^8^	Not observed	10–20^9^	Not observed
terminal t_1/2_, h	1.42	5.3	7.4 / 9.4	27–30^10^	14.7
C_max_, mg Fe/L	20.6	35.3	37 / 331	-	130
AUC, mg Fe/L*h	43.7	83.3	333 / 6,277	6,853^11^	2,912
AUC, standardized for a dose of 100 mg Fe, mg Fe/L*h	35.0	83.3	333 / 627.7	1,371	922
MRT, h		5.5	11.2 / 16.5	-	-
CL, L/h	2.99	1.23	0.26 / 0.16	-	0.11
V_c_, L	6.02	3.2	2.7 / 2.1	3.0	2.3
Fe-transport, mg Fe/day	Not calculated	31.0	Not accessible	240–480^9^	Not accessible

PK, pharmacokinetics; k_el_, the first-order rate constant for elimination; k_0_, the zero-order rate constant for elimination; t_1/2_, half-life; C_max_, peak concentration; AUC, area under the curve; MRT, mean residence time; CL: clearance; V_c_, initial distribution volume

* Injectafer^®^ in some markets;

1 Method according to USP Iron sucrose injection, relative to a pullulan standard; also published by Geisser et *al.* 1992 [17];

2 Method according to Balakrishnan *et al.* 2009 [23], relative to a protein standard;

3 Seligman *et al.* 2004 [21]: Study in iron deficient subjects;

4 Danielson *et al.* 1996 [20]: Study in healthy volunteers;

5 Geisser *et al.* 2010 [22]: Study in volunteers with mild iron deficiency anemia;

6 Henderson *et al.* 1969 [24]: Study in iron deficient patients;

7 Landry *et al.* 2005 [25]: Study in normal subjects and hemodialysis patients;

8 Elimination due to transferrin binding;

9 Elimination due to reaction with macrophages/RES at doses above 500 mg iron;

10 Calculated from Figure 5 and Figure 6 in Henderson *et al.* 1969 [24];

11 Calculated for a dose of 500 mg iron by using t_1/2_ (terminal k_el_) and V_d_
